# Prevalence and risk factors for colorectal polyps in a Chinese population: a retrospective study

**DOI:** 10.1038/s41598-020-63827-6

**Published:** 2020-04-24

**Authors:** Jiaqi Pan, Li Cen, Lei Xu, Min Miao, Youming Li, Chaohui Yu, Zhe Shen

**Affiliations:** 1 Department of Gastroenterology, The First Affiliated Hospital, College of Medicine, Zhejiang University, Hangzhou, China; 2 Department of Gastroenterology, Ningbo First Hospital, Ningbo, China; 3 Department of Gastroenterology, The Affiliated Hospital of Ningbo University, School of Medicine, Ningbo, China

**Keywords:** Colonoscopy, Gastrointestinal diseases, Gastroenterology, Risk factors

## Abstract

The incidence of colorectal polyps is rising. Certain types of polyps are considered to be the precursor lesions for colorectal cancers. To investigate the prevalence and related factors of colorectal polyps in Chinese subjects, we first performed a cross-sectional study. A total of 3066 subjects were documented, and the prevalence of colorectal polyps was 18.1%. Then we evaluated the incidence and risk factors of polyps via a retrospective cohort study in the same population. 561 subjects who received at least twice surveillance colonoscopies with available reports during the study period and had no polyp at the first endoscopy were included in the retrospective cohort study, of whom 19.1% developed colorectal polyps. Regular smoking was independently associated with the presence and development of colorectal polyps. Further analyses indicated that polyps were associated with smoking status, daily cigarette consumption, and drinking habit. Moreover, smoking tends to be more relavent to rectal, small and single polyp. In conclusion, colorectal polyp is a common disease in China. Exploring the epidemiology and risk factors may improve the prevention of colorectal polyps, even colorectal cancer.

## Introduction

The incidence of colorectal polyp is rapidly increasing worldwide. Colorectal cancer (CRC) was found to be the third most common cancer among men and the fourth most common cancer among women^1^. Adenomatous polyps are thought to be the precursor lesions for the majority of CRCs^2^, developing mostly through an adenoma-carcinoma sequence^3^. Recent studies proposed that hyperplastic polyps also contribute to CRCs through serrated or microsatellite instable pathways^4,5^. Some polyps may cause gastrointestinal symptoms such as hematochezia, stomachache, abdominal distension, affecting health and quality of life. More seriously, other asymptomatic polyps may quietly develop into malignancies, which are more likely to be ignored. The detection and resection of precancerous polyps under colonoscopy is a critical way to reduce the incidence of CRC and its subsequent morbidity and mortality^6,7^. However, to explore the prevalence and risk factors for colorectal polyps could be a better way to prevent and manage this disease in advance.

Population characteristics, living habits, and health conditions are closely related to the presence and development of colorectal polyps and cancers. In recent studies, some risk factors for adenomatous polyps were reported, including sex, metabolic syndrome, *Helicobacter pylori* infection, smoking, alcohol drinking, etc.^8–10^. As for CRC, a population-based colorectal cancer screening program in China suggested that age, gender, BMI, family history, meat intake and smoking were associated with colorectal neoplasms^11^. Smoking is proposed to be closely associated with colorectal polyps, neoplasia, and CRCs^11–13^. However, most evidence came from case-control or cross-sectional studies, and there is still no cohort study to evaluate the risk factors for colorectal polyps in China.

Therefore, we first evaluated the prevalence of colorectal polyps and factors associated with the presence of this disease in a cross-sectional study. Then we investigated the incidence and risk factors for the colorectal polyps via a retrospective cohort study. Associations between the risk factors and features of colorectal polyps were further assessed.

## Methods

We designed two linked studies to investigate the prevalence and incidence of colorectal polyps, and risk factors for its presence and development in a Chinese population. The first was a cross-sectional study to determine the prevalence of colorectal polyps and factors associated with its presence. Then we conducted a retrospective cohort study of baseline polyp-free subjects from the first study who underwent at least twice surveillance colonoscopies to assess the incidence and risk factors for colorectal polyps.

### Study population

**Figure 1:**
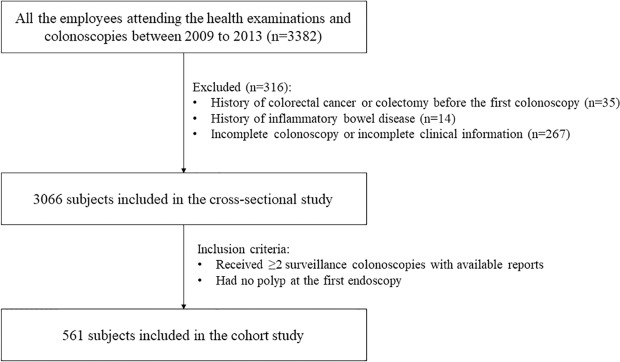
Flowchart of subject selection.

This retrospective study was performed among the employees of Zhenhai Refining & Chemical Company (Ningbo, China). All of the employees attending the health examinations and colonoscopies between January 2009 to December 2013 were screened for enrollment. Exclusion criteria were: (1) history of colorectal cancer or colectomy before the first surveillance colonoscopy; (2) history of inflammatory bowel disease; (3) incomplete colonoscopy or incomplete clinical information. A total of 3066 eligible subjects were enrolled in the cross-sectional study to investigate the prevalence of colorectal polyp and potential factors associated with its presence. Then in the same population, subjects who had at least twice surveillance colonoscopies and had no polyp at the first endoscopy were selected for the retrospective cohort study (Fig. 1).

### Data collection

All of the data were collected by reviewing the medical records of the medical center. The procedures of health examination were the same as those used at baseline during the study period. Height and weight were measured on standardised machines. Subjects were stratified to three groups according to BMI: underweight (BMI < 18.5), normoweight (18.5 ≤ BMI < 25) and overweight (BMI ≥ 25). Blood samples were analysed in the same laboratories. In blood tests, following data were collected: white blood cell (WBC) count, red blood cell (RBC) count, platelet, hemoglobin, plasma viscosity, erythrocyte sedimentation rate (ESR), albumin, total cholesterol (TC), triglycerides (TG), creatinine and uric acid.

Smoking and drinking habits, medical histories such as colorectal cancer, colectomy, and inflammatory bowel disease were inquired by a physician. Regular smoking (or current smoking) was defined as smoking at least one cigarette per day. Former smoking status was defined as a cessation of smoking for at least one year. Regular drinking was defined as drinking at least one alcoholic beverage per week.

For each colonoscopy report, we extracted the following data: date, quality of bowel preparation, presence of polyps, diameter of the biggest polyp, position and number of the polyps. Duration of follow-up was defined as the interval time between baseline and the examination when colorectal polyps were first identified, or the last endoscopy before the end of our study period.

### Statistical analysis

The statistical analyses were conducted using SPSS 24.0 (SPSS, Chicago, IL, USA). Continuous variables were presented as mean with standard vision or medians with interquartile range, and were compared by Student’s *t*-test or Mann-Whitney *U* test. Categorical variables were expressed as frequencies with percentages and were analysed by chi-squared(χ^2^) test. Logistics regression was used to evaluate the factors associated with the presence of colorectal polyps in the cross-sectional study. Cox proportional hazards regression analyses were applied to assess the risk factors for the development of colorectal polyps in the retrospective cohort study. Those considerable covariates (p < 0.10 or have important clinical significance) were included in multivariate analysis. Interactions among the covariates in multivariate analyses were tested by collinearity diagnosis; no significant collinearity was found. A *P* value < 0.05 (two-tailed) was considered to be statistically significant.

### Ethics

This study was performed according to the Declaration of Helsinki. The study protocol was approved by the Research Ethics Committee of the First Affiliated Hospital, College of Medicine, Zhejiang University (Reference Number: 2019648). Due to the retrospective nature of the study, informed consent was waived.

## Results

### Characteristics of subjects in the cross-sectional study

A total of 3066 subjects were documented in the cross-sectional study (Fig. 1), of whom 554 (18.1%) had colorectal polyps. The subjects with colorectal polyps tend to be older, male, regular smoker and drinker, and they had higher WBC and RBC counts, hemoglobin, plasma viscosity, creatinine, uric acid, and lower platelet (Table 1). There was no difference between the parameters of lipid metabolism TC and TG.

**Table 1:** Characteristics of subjects in the cross-sectional study.

Variables	With colorectal polyp (n = 554)	Without colorectal polyp (n = 2512)	*P*
Age (years)	60 (53–67)	55 (47–62)	<0.001
Gender (male/female, n)	395/159	1461/1051	<0.001
**Body mass index** (**kg/m**^**2**^**)**			
18.5~25	361 (65.3)	1711 (68.3)	1 (reference)
<18.5	16 (2.9)	78 (3.1)	0.92
≥25	176 (31.8)	715 (28.6)	0.131
Total cholesterol (mmol/L)	5.07 (4.53–5.70)	5.00 (4.44–5.66)	0.083
Triglycerides (mmol/L)	1.20 (0.87–1.74)	1.18 (0.84–1.70)	0.26
Regular smoker (%)	158 (28.5)	514 (20.5)	<0.001
Regular drinker (%)	125 (22.6)	397 (15.9)	<0.001
White blood cell count (×10^9^/L)	5.85 (4.98–6.80)	5.60 (4.80–6.60)	0.005
Red blood cell count (×10^12^/L)	4.59 (0.45)	4.55 (0.45)	0.037
Platelet (×10^9^/L)	200 (169–230)	205 (175–242)	0.005
Hemoglobin (g/L)	140 (130–148)	137 (127–147)	0.001
Plasma viscosity (mpa.s)	1.38 (1.32–1.41)	1.37 (1.31–1.40)	0.001
Erythrocyte sedimentation rate (mm/h)	8 (4–14)	9 (4–14)	0.299
Albumin (g/L)	44.80 (43.30–46.50)	45.00 (43.50–46.60)	0.123
Creatinine (umol/L)	63 (54–72)	62 (52–71)	0.024
Uric Acid (umol/L)	321 (267–385)	312 (258–371)	0.006

### Factors associated with the presence of colorectal polyps

Logistic regression analysis was applied to evaluate the factors associated with the presence of colorectal polyps (Table 2). In the univariate model, 12 variables were potentially correlated with the presence of colorectal polyps, which were entered into the multivariable regression analysis. The results indicated that older age, male, regular smoking, higher TC and WBC count were significantly associated with the presence of colorectal polyps.

**Table 2:** Factors associated with the presence of colorectal polyps, logistic regression analysis.

Variables	Mean (s.d.) or median (IQR) or number(%)	Univariable model		Multivariate model^a^	
		HR (95%CI)	*P* value	HR (95%CI)	*P* value
Age (years)	56 (47–63)	1.037 (1.028–1.046)	<0.001	1.039 (1.029–1.049)	<0.001
Gender (male/female, n)	1856/1210	1.787 (1.463–2.184)	<0.001	1.545 (1.119–2.134)	0.008
**Body mass index** (**kg/m**^**2**^**)**					
18.5~25	2072 (67.8)	1 (reference)			
<18.5	94 (3.1)	0.972 (0.561–1.685)	0.92		
≥25	891 (29.1)	1.167 (0.955–1.425)	0.131		
Total cholesterol (mmol/L)	5.02 (4.45–5.66)	1.089 (0.988–1.202)	0.087	1.140 (1.025–1.266)	0.015
Triglycerides (mmol/L)	1.18 (0.84–1.71)	1.069 (0.976–1.171)	0.153		
Regular smoker (%)	672 (21.9)	1.546 (1.255–1.904)	<0.001	1.351 (1.050–1.738)	0.019
Regular drinker (%)	522 (17.1)	1.547 (1.234–1.940)	<0.001	1.292 (0.999–1.672)	0.051
White blood cell count (×10^9^/L)	5.7 (4.80–6.60)	1.092 (1.027–1.160)	0.005	1.076 (1.003–1.154)	0.041
Red blood cell count (×10^12^)	4.55 (0.45)	1.240 (1.013–1.517)	0.037	1.038 (0.760–1.417)	0.816
Platelet (×10^9^/L)	204 (174–239)	0.997 (0.996–0.999)	0.006	0.998 (0.996–1.001)	0.145
Hemoglobin (g/L)	138 (127–147)	1.012 (1.005–1.019)	0.001	0.998 (0.986–1.010)	0.777
Plasma viscosity (mpa.s)	1.37 (1.31–1.41)	5.432 (1.796–16.426)	0.003	1.040 (0.273–3.960)	0.954
Erythrocyte sedimentation rate (mm/h)	8 (4–14)	0.996 (0.984–1.008)	0.996		
Albumin (g/L)	44.90 (43.50–46.60)	0.970 (0.933–1.009)	0.131		
Creatinine (umol/L)	62 (53–71)	1.010 (1.004–1.017)	0.002	1.003 (0.998–1.009)	0.275
Uric Acid (umol/L)	314 (260–373)	1.001 (1.000–1.003)	0.011	0.999 (0.997–1.000)	0.084

^a^Adjusted for age, gender, total cholesterol, regular smoker, regular drinker, white blood cell count, red blood cell count, platelet, hemoglobin, plasma viscosity, creatinine, uric acid.

### Baseline characteristics of subjects in the cohort study

A total of 561 subjects (338 males and 223 females) who had no colorectal polyps at baseline and underwent at least twice surveillance colonoscopies were included in the cohort study. The average follow-up time was 3.42 years. Baseline characteristics of 561 subjects were presented in Table 3. In this cohort, subjects who developed colorectal polyps were more likely to be male, regular smokers, and had higher BMI, WBC and RBC counts, hemoglobin, plasma viscosity, creatinine, and uric acid at baseline. Besides, the subjects with colorectal polyps had lower TC, albumin, and ESR. Age, TG, regular drinking and platelet were not significantly different between the two groups.

**Table 3:** Characteristics of the subjects at baseline in the cohort study.

Variables	With colorectal polyps (n = 107)	Without colorectal polyp (n = 454)	*P*
Age (years)	59 (56–65)	60 (55–67)	0.898
Gender (male/female, n)	83/24	255/199	<0.001
**Body mass index** (**kg/m2)**			
18.5~25	61 (57.0)	302 (66.7)	1 (reference)
<18.5	2 (1.9)	12 (2.6)	0.804
≥25	44 (41.1)	139 (30.6)	0.043
Total cholesterol (mmol/L)	4.97 (0.94)	5.16 (0.91)	0.044
Triglycerides (mmol/L)	4.84 (4.40–5.68)	5.15 (4.55–5.70)	0.489
Regular smoker (%)	32 (29.9)	52 (11.5)	<0.001
Regular drinker (%)	30 (28.0)	93 (20.5)	0.089
White blood cell count (×10^9^/L)	5.9 (4.8–6.7)	5.3 (4.5–6.4)	0.019
Red blood cell (×10^12^)	4.59 (0.41)	4.44 (0.43)	0.002
Platelet (×10^9/L)	201.7 (47.8)	205.8 (46.0)	0.411
Hemoglobin (g/L)	142.0 (134–151)	137.0 (126.0–147.0)	<0.001
Plasma viscosity (mpa.s)	1.38 (1.33–1.42)	1.36 (1.31–1.41)	0.029
Erythrocyte sedimentation rate (mm/h)	7.0 (4.0–12.0)	9.0 (5.0–14.0)	0.001
Albumin (g/L)	44.17 (2.160)	44.68 (1.981)	0.019
Creatinine (umol/L)	68.1 (14.5)	62.5 (13.6)	<0.001
Uric Acid (umol/L)	348.0 (89.2)	324.8 (83.2)	0.011

### Risk factors for the development of colorectal polyps

During the 1919 person-years of follow-up, 107 (19.1%) subjects developed colorectal polyps. Cox proportional hazards regression analysis was applied to explore the factors associated with the development of colorectal polyps (Table 4). In the univariate models, 5 variables were correlated with the development of colorectal polyps: gender, regular smoking, red blood cell, hemoglobin, and creatinine. While in the multivariate models, regular cigarette smoking (adjusted hazard ratio [AHR] 1.842; 95% CI 1.139–2.980; *P* = 0.013) and lower albumin (AHR 0.901; 95% CI 0.819–0.991; *P* = 0.032) were the independent risk factors for the development of colorectal polyps.

**Table 4:** Risk factors for the development of colorectal polyps, Cox regression analysis.

Variables	Mean (s.d.) or median (IQR) or number(%)	Univariable model		Multivariate model^a^	
		HR (95%CI)	*P* value	HR (95%CI)	*P* value
Age (years)	60.0 (55.0–66.5)	0.993 (0.970–1.016)	0.543	0.994 (0.969–1.020)	0.658
Gender (male/female, n)	338/223	1.669 (1.057–2.636)	0.028	0.869 (0.440–1.717)	0.686
**Body mass index, n** (**%)**					
18.5~25	363 (64.8)	1 (reference)		1	
<18.5	14 (2.5)	0.723 (0.177–2.957)	0.652	1.001 (0.239–4.186)	0.999
≥25	183 (32.6)	1.414 (0.959–2.083)	0.08	1.255 (0.841–1.872)	0.266
Total cholesterol (mmol/L)	5.13 (0.92)	0.869 (0.702–1.076)	0.197		
Triglycerides (mmol/L)	1.25 (0.89–1.81)	0.898 (0.733–1.101)	0.301		
Regular smoker (%)	83 (14.8)	2.150 (1.420–3.254)	<0.001	1.842 (1.139–2.980)	0.013
Regular drinker (%)	123 (21.9)	1.191 (0.780–1.816)	0.418	0.812 (0.508–1.299)	0.385
White blood cell count (×10^9^/L)	5.4 (4.6–6.5)	1.081 (0.965–1.211)	0.178		
Red blood cell (×10^12^)	4.47 (0.43)	1.681 (1.062–2.661)	0.027	1.174 (0.516–2.669)	0.702
Platelet (×10^9^/L)	205.0 (46.4)	0.998 (0.994–1.002)	0.282		
Hemoglobin (g/L)	138.0 (128.0–147.0)	1.022 (1.007–1.038)	0.005	1.018 (0.988–1.048)	0.244
Plasma viscosity (mpa.s)	1.37 (1.31–1.41)	3.185 (0.342–29.692)	0.309		
Erythrocyte sedimentation rate (mm/h)	9.0 (5.0–14.0)	0.976 (0.945–1.007)	0.127		
Albumin (g/L)	44.59 (2.024)	0.921 (0.839–1.010)	0.081	0.901 (0.819–0.991)	0.032
Creatinine (umol/L)	63.6 (13.9)	1.015 (1.001–1.028)	0.03	1.009 (0.992–1.027)	0.277
Uric Acid (umol/L)	329.2 (84.8)	1.001 (0.999–1.003)	0.246		

^a^Adjusted for age, gender, body mass index, regular smoker, regular drinker, red blood cell count, hemoglobin, plasma viscosity, albumin, creatinine.

### Further analysis between smoking and colorectal polyps

We further explored the association between cigarette smoking ways and colorectal polyp features in different regression models (Table 5). After adjusted for major confounding factors, the risk for colorectal polyps in current smokers was significantly higher than that in never-smokers (AHR 1.786; 95%CI 1.087–2.936; *P* = 0.022). People who smoked more than 20 cigarettes per day were more likely to develop colorectal polyps (AHR 1.878; 95%CI 1.018–3.463; *P* = 0.044) than those who smoked less (AHR 1.811; 95%CI 1.003–3.270; *P* = 0.049). Since smoking and drinking are always mentioned together, we found that subjects with both smoking and drinking habits had a significantly higher risk for colorectal polyps (AHR 2.073; 95%CI 1.196–3.593; *P* = 0.009).

**Table 5:** Associations between different smoking ways and the development of colorectal polyps.

	n (%)	Model 1^a^		Model 2^b^		Model 3^c^	
		HR (95%CI)	*P* value	HR (95%CI)	*P* value	HR (95%CI)	*P* value
**Smoking status**							
Never	446 (79.5)	1		1		1	
Former	32 (5.7)	1.070 (0.465–2.465)	0.873	0.949 (0.404–2.229)	0.904	0.817 (0.344–1.940)	0.647
Current	83 (14.8)	2.161 (1.420–3.289)	<0.001	1.914 (1.187–3.086)	0.008	1.786 (1.087–2.936)	0.022
**Cigarettes per day**							
0	478 (85.2)	1		1		1	
1–20	44 (7.8)	2.175 (1.267–3.733)	0.005	1.950 (1.099–3.459)	0.022	1.811 (1.003–3.270)	0.049
>20	39 (7.0)	2.125 (1.238–3.648)	0.006	1.904 (1.059–3.426)	0.032	1.878 (1.018–3.463)	0.044
**Smoke and drink**							
no smoking and drinking	478 (85.2)	1		1		1	
Both smoke and drink	41 (7.3)	2.525 (1.540–4.138)	<0.001	2.263 (1.325–3.865)	0.003	2.073 (1.196–3.593)	0.009

^a^Unadjusted.

^b^Adjusted for age and gender.

^c^Adjusted for age, gender, body mass index, regular smoker, regular drinker, red blood cell count, hemoglobin, plasma viscosity, albumin, creatinine.

The relation between smoking and colorectal polyps was different in polyp position, size and number (Table 6). Regular smoking induced a higher risk for rectal polyps (AHR 2.975; 95%CI 1.315–6.726; *P* = 0.009) rather than colonic polyps (AHR 1.544; 95%CI 0.877–2.721; *P* = 0.133). Besides, smoking tends to be associated with small (<1 cm) polyps (AHR 1.903; 95%CI 1.154–3.136; *P* = 0.012) and single polyp (AHR 2.112; 95% CI 1.182–3.773; *P* = 0.012).

**Table 6:** Associations between cigarette smoking and colorectal polyp features.

Variable	n (%)	Model 1^a^		Model 2^b^		Model 3^c^	
		HR (95%CI)	*P* value	HR (95%CI)	*P* value	HR (95%CI)	*P* value
**Polyp position**							
colon	81 (75.7)	1.836 (1.107–3.045)	0.019	1.587 (0.913–2.758)	0.102	1.544 (0.877–2.721)	0.133
rectum	32 (29.9)	3.985 (1.966–8.075)	<0.001	3.543 (1.579–7.948)	0.002	2.975 (1.315–6.726)	0.009
**Maximum polyp size**							
<1 cm	101 (94.4)	2.186 (1.425–3.352)	<0.001	1.970 (1.218–3.185)	0.006	1.903 (1.154–3.136)	0.012
≥1 cm	6 (5.6)	3.174 (0.581–17.349)	0.183	2.458 (0.400–15.114)	0.332	2.348 (0.365–15.127)	0.369
**Number of polyp**(**s)**							
1	72 (67.3)	2.485 (1.504–4.106)	<0.001	2.200 (1.253–3.863)	0.006	2.112 (1.182–3.773)	0.012
≥2	35 (32.7)	2.297 (1.103–4.788)	0.026	2.093 (0.928–4.719)	0.075	1.875 (0.820–4.287)	0.136

^a^Unadjusted.

^b^Adjusted for age and gender.

^c^Adjusted for age, gender, body mass index, regular smoker, regular drinker, red blood cell count, hemoglobin, plasma viscosity, albumin, creatinine.

## Discussion

We performed two linked studies to assess the prevalence and risk factors for the presence and development of colorectal polyps. In the cross-sectional study, the prevalence of colorectal polyps was 18.1% in our study population. Age, gender, TC, regular smoking and WBC count were independently associated with the presence of colorectal polyps. The retrospective cohort study revealed that the cumulative incidence of subjects developed colorectal polyps was 19.1% during their follow-up colonoscopy surveillance. Regular cigarette smoking and albumin were independent risk factors for the development of colorectal polyps. Thus the two studies have the consistent result that smoking plays an essential role in the presence and development of colorectal polyps. Further analyses showed those who were current smokers, had more daily smoking consumption, and combined with regular drinking had a higher risk of developing colorectal polyps. Moreover, regular smoking was more relevant to rectal, small (<1 cm) and single polyp.

In recent years, the prevalence of colorectal polyps is increasing, which may be influenced by factors like changes in diet and lifestyle habits. Moreover, another important reason is that the improved equipment or colonoscopy techniques cause higher detection rates over time^14^. The incidence of colorectal polyp and CRC varies in different geographical locations and races. The highest incidence is found in western countries, while the lowest is in Africa and South-Central Asia^15^. A large study from the United States reported that 44.9% polypectomies were performed on 17275 patients who underwent average-risk screening colonoscopy between 2005–2006^16^. However, endoscopy is much more accessible in western countries. Besides, interval CRC risk was found higher in blacks than in whites^17^. Recently, there are more Asian studies investigating prevalence and risk factors for colorectal polyps and CRC. A Korean study reported that the prevalence of colorectal adenomas was 34.5% in men and 20.0% in women among subjects at average risk, and the prevalence increased annually over the study period^14^. A Thai study found that in the population aged 50–65 years old, 18.2% of subjects had adenomatous polyps, of which 7% were high-risk adenoma^18^. In our study, the prevalence of colorectal polyp was 18.1% at baseline, while almost one-fifth subjects developed colorectal polyps in the subsequent cohort study. Therefore, it is crucial to investigate the risk factors of this disease.

Smoking is a well-known modifiable risk factor for colorectal polyps and CRC^19–22^. We found that regular cigarette smoking is an independent risk factor for the presence and development of colorectal polyps in Chinese population. Previous studies have revealed dose-response relations among the daily number of cigarettes smoked, the duration of smoking, the pack-years of smoking, and the risk for colorectal polyps^23,24^. The association was robust in all kinds of polyps (sessile serrated polyps, conventional adenomas, and hyperplastic polyps). Reduced risk of all stages of colorectal carcinogenesis (hyperplastic polyps, non-advanced adenomas, and advanced CRN) was found in people with a healthy lifestyle, including nonsmoking^25^. Previous studies revealed some potential mechanisms for the association between smoking, colorectal polyps and CRN, such as the reduced methylation of relevant genes^26^, genetic variants in carcinogen-metabolising enzymes^12^, the polymorphisms in DNA repair genes EXO1 and ATM^27^, the mutations in mismatch repair enzymes^28^, and XPC polymorphisms^29^, etc. In a word, tobacco contains many carcinogens that are thought to create no less than irreversible genetic damage to the colorectal mucosa, initiating the formation of colorectal polyps^19^.

It’s worth mentioning that drinking and smoking often together affect the prevalence, occurrence, and development of many diseases. In 2007, the International Agency for Research on Cancer (IARC) indicated that there was sufficient evidence to support the inclusion of CRC in the list of alcohol-related malignancies^30,31^. Recent meta-analysis showed that alcohol also linked with an increased risk of colorectal adenomas^32^ and serrated polyps^33^. However, the association appears to be more influential in European studies compared to those conducted in Asia or the US^32^. Our study failed to show the effect of alcohol alone on the development of colorectal polyps. Possible reasons could be genetic and lifestyle differences. Nevertheless, we found that when drinking combined with a regular smoking, the risk for colorectal polyp was doubled. This phenomenon may be due to the interactive effects of alcohol and tobacco/nicotine on cross-cue reactivity to alcohol and tobacco craving, subjective feelings of stimulation and sedation, and alcohol and smoking self-administration^34^. In addition, smoking and alcohol could cause changes in the gastrointestinal microbiome^35^. However, specific mechanisms need to be further explored.

The current study also demonstrated that regular tobacco consumption tends to cause rectal, small and single polyps. Consistent with our result, a stronger association between smoking and distal rather than proximal polyps was also found in another study^36^. This phenomenon might be explained that distal colorectal carcinogenesis is highly associated with environmental carcinogens such as various chemicals while proximal carcinogenesis is more relevant to genetic background^37,38^. A case-control study from Germany suggested that frequent cigarette smoking was an independent risk factor for the occurrence of large (≥20 mm) colorectal polyps, but our results showed a more substantial relation to small (<10 mm) polyps, possibly due to the small number of the subjects with large polyps in our study, as well as the racial and genetic disparities^39^. Nevertheless, the size and number of polyps are changing with the prolonging of time. Within the time frame of our study, smoking tends to be associated with rectal, small and single polyps.

As is known to all, diseases would result in nutrient consumption. Meanwhile, malnutrition is also a significant problem that could cause a number of clinical consequences in turn, such as deteriorated quality of life, decreased response to treatment, and shorter survival rate^40^. Serum albumin is a recognised biomarker for assessing the nutritional condition and were considered to be an important factor to predict recovery and survival of CRC^41^. In our study, decreased albumin was found to be associated with the development of colorectal polyps. In a recent study, Sun F *et al*. found elevated FAR (FAR = 100*Fibrinogen/Albumin) in newly diagnostic CRC patients compared with the healthy or benign controls, while albumin was low in the cases^42^. However, the specific association between albumin and the development of colorectal polyps remains to be further studied.

Age and gender might be unmodifiable factors for polyps. The prevalence of colorectal polyps and CRC generally increases with age^18^. Our results also showed increasing age associated with the presence of colorectal polyps. Most studies reported a higher prevalence in men than in women, which even can’t be explained by the influence of cardiovascular and lifestyle risk factors^43^. A cross-sectional analysis of the data from a large colonoscopy-based screening program showed that in each age group, the numbers needed to undergo colorectal-cancer screening for detecting advanced neoplasia was significantly lower in men than in women^44^. However, we didn’t find relevance between sex and colorectal polyps after adjusted for confound factors, but the trend existed in the first study.

Some factors were found not significantly associated with colorectal polyps in multivariable analysis, such as BMI, lipid metabolic parameters, WBC and RBC. Previous studies indicated a weak association between BMI^45,46^, serum lipids^47^ and colorectal polyps, but not all^48^. Mechanisms related to insulin resistance and inflammation have been postulated to be involved in colorectal carcinogenesis. In our study, TC and WBC were related to the presence of polyps, while others did not independently influence the presence and development of colorectal polyps, but modest relevance could not be excluded.

The present study had some limitations that should be acknowledged. First, as is a retrospective study, we failed to get sufficient polyp histopathology reports of every subject to further evaluate the risk factors for different pathological types of colorectal polyps. Second, data on smoking and drinking were self-reported, which may cause recall bias. Last, we did not explore the mechanism of how smoking and decreased albumin increases the risk of polyps. Future studies are expected to clarify the molecular mechanisms and signal pathways of these factors in the development of colorectal polyps.

In summary, our study indicated that colorectal polyps are prevalent in China, and nearly one-fifth subjects developed polyps during the study period. Smoking was significantly associated with the presence and development of polyps, especially related to the rectal, small and single polyp. The incidence of colorectal polyps was also influenced by smoking status, daily tobacco consumption, and whether smoking was combined with a drinking habit. As potential precursor lesions of CRC, we should pay more attention to the risk factors for colorectal polyps, to better prevent and manage this series of disease.
